# Reply: ‘Childhood leukaemia and socioeconomic status’

**DOI:** 10.1038/bjc.2012.171

**Published:** 2012-06-12

**Authors:** M E Kroll, C A Stiller, M F G Murphy

**Affiliations:** 1 Childhood Cancer Research Group, University of Oxford, Richards Building, Oxford OX3 7LG, UK; 2 Cancer Epidemiology Unit, University of Oxford, Richard Doll Building, Oxford OX3 7LF, UK

**Sir,**

We thank you for giving us the opportunity to respond to Dr Franceschi’s (2012) letter.

We reported consistently higher childhood leukaemia incidence rates in more affluent communities within England and Wales in each of the three decades up to 2005, and discussed several possible explanations (Kroll *et al*, 2011). Dr Franceschi queries our interest in the possibility of uneven diagnosis, and suggests that it would be of interest if the effects of adjustment by maternal parity and/or maternal age could be reported.

It is true that the relationship of childhood leukaemia to the socioeconomic measure used in our study (quintiles of the Carstairs deprivation index) might have been attenuated if the analysis had been adjusted by maternal parity and/or maternal age (or any other factor related to socioeconomic status). We were unable to make such adjustments because this was a study of incidence in the whole childhood population, not a case–control study, and birth records were not available for all registered cases. However, we note that, strictly, these factors are not ‘known to influence childhood leukaemia risk’, as Dr Franceschi implies, but are known to be associated with it; the explanation is unknown.

The study mentioned by Dr Franceschi (Dockerty *et al*, 2001) was included in the systematic review that we cited (Poole *et al*, 2006), and was therefore not discussed individually in our paper. This study differed from ours in several respects. It was a case–control study for the diagnosis period 1968–1986, restricted to children for whom birth records were available, and using a deprivation score derived from address at birth, rather than address at diagnosis. Nevertheless, with increasing deprivation there was a statistically significant decreasing trend in risk of acute lymphoblastic leukaemia, the major subtype in children (Table 5, Dockerty *et al*, 2001).

We certainly did not mean to suggest that British paediatricians discriminate in any way in the care they provide. Rather, we suggest that recognition of leukaemia as a potential underlying cause of non-specific symptoms might be uneven: for example, in poorer communities, provision of primary care may be less generous, and parents may be younger and less well-educated. Thus, for example, under-diagnosis might contribute to the associations with maternal parity and maternal age that Dr Franceschi mentions. A further study (Kroll *et al*, 2012) uses clinical data to examine this possibility in detail.
